# Vaccination of Chickens with SPI1-*lon* and SPI1-*lon*-*fliC* Mutant of *Salmonella enterica* Serovar Enteritidis

**DOI:** 10.1371/journal.pone.0066172

**Published:** 2013-06-13

**Authors:** Marta Matulova, Hana Havlickova, Frantisek Sisak, Ivan Rychlik

**Affiliations:** Veterinary Research Institute, Brno, Czech Republic; Institut National de la Recherche Agronomique, France

## Abstract

The prevalence of *Salmonella enterica* serovar Enteritidis is gradually decreasing in poultry flocks in the EU, which may result in the demand for a vaccine that allows for the differentiation of vaccinated flocks from those infected by wild-type *S*. Enteritidis. In this study, we therefore constructed a (Salmonella Pathogenicity Island 1) SPI1-*lon* mutant with or without *fliC* encoding for *S*. Enteritidis flagellin. The combination of SPI1-*lon* mutations resulted in attenuated but immunogenic mutant suitable for oral vaccination of poultry. In addition, the vaccination of chickens with the SPI1-*lon*-*fliC* mutant enabled the serological differentiation of vaccinated and infected chickens. The absence of *fliC* therefore did not affect the immunogenicity of the vaccine strain and allowed for serological differentiation of the vaccinated chickens. The SPI1-*lon*-*fliC* mutant is therefore a suitable marker vaccine strain for oral vaccination of poultry.

## Introduction

*Salmonella enterica* serovar Enteritidis (*S*. Enteritidis) colonises chickens usually without any gross clinical signs, however, inflammation can be recorded in the intestinal tract, caecum in particular [1]–[3]. Susceptibility of chickens to *S*. Enteritidis decreases with age and 6 week old chickens are usually quite resistant to *S*. Enteritidis infection [4], [5]. The prevalence of *S*. Enteritidis in poultry flocks is gradually decreasing in the EU member states [6]. One of the reasons for such a decrease is the use of vaccination in egg producing flocks, usually with live, attenuated *Salmonella* vaccines. Current commercial vaccines are therefore of great importance in *Salmonella* control programs. However, with a decreasing prevalence, the demand for a simple differentiation of vaccinated flocks from those infected by wild-type *S*. Enteritidis will increase and this is something that the current commercial vaccines cannot provide.

Several laboratories therefore initiated research on a *Salmonella* marker vaccine [7], [8]. In our previous study, we showed that deletion of the *fliC* gene from *S*. Enteritidis might be an interesting option of how to construct a marker vaccine [9]. This genetic modification has a considerable advantage when compared with other approaches since there is a commercially available ELISA kit detecting the presence of anti-*S*. Enteritidis flagellin antibodies in chicken serum. However, flagellin is also one of the pathogen associated molecular patterns recognized by TLR5 [10], [11]. In agreement with this, the deletion of flagella in *S*. Typhimurium led to its less efficient recognition by the host immune system and a temporary increase in the virulence in the early stages of chicken infection [12]. On the other hand, over-expression of flagella resulted in a lower invasiveness of *S*. Enteritidis, perhaps due to efficient TLR5 dependent recognition of the vaccine [13]. Due to the dual role of flagella both as a major T and B cell antigen and pathogen associated molecular pattern, there are therefore concerns that if a *Salmonella* vaccine strain stimulates the production of anti-flagella antibodies, these may then bind to flagella expressed by the invading wild-type *S*. Enteritidis and interfere with its correct recognition by TLR5, as has been shown in *E*. *coli* in cattle [14]. On the other hand, an aflagellated vaccine not inducing anti-flagella antibodies may allow for the efficient recognition of challenge *Salmonella* by innate TLR5-dependent recognition and specific immunity against all remaining *Salmonella* antigens, as observed in *S*. *enterica* immunized and challenged mice [7], [15].

The virulence of *Salmonella enterica* can be attenuated by many different approaches. By understanding the function of type III secretion systems encoded by two different pathogenicity islands, SPI1 and SPI2, the mutants disabled in these virulence factors were constructed and used as live, attenuated vaccines. Interestingly, whilst SPI2 mutants of *S*. *enterica* are attenuated in all warm-blooded hosts, SPI1 mutants seem to be attenuated only in hosts for which an enteric type of disease is characteristic and these genes are dispensable when the output of the infection is a typhoid disease [16]–[18]. In agreement with the previous statement, the removal of SPI1 genes from *S*. Enteritidis or *S*. Typhimurium, i.e. the serovars which cause a mild enteric disease in chickens, results in a decrease in virulence with preserved immunogenicity in these hosts [5], [19], [20]. Moreover, SPI1 mutants are defective in early interactions with macrophages which may enable the macrophage’s proper antigen processing and presentation [21]–[23] though the role of SPI1 in the interactions with other antigen presenting cells in the chicken is less clear. This may finally result in an efficient specific immune response, as we have shown recently [5].

The above-mentioned results suggest that the ΔSPI1-*fliC* mutant might be an interesting vaccine strain because it should be attenuated in virulence and also should enable serological differentiation of vaccinated and infected chickens. However, given the concerns on increased virulence of flagella defective mutants [12], we were thinking of additional independent attenuation. One of the possibilities was the inactivation of gene encoding Lon protease what results in a mucoid colony phenotype [24]. Lon protease also is a negative regulator of SPI1 genes [25] and is required for the resistance to multiple environmental stresses [26]. We have shown earlier that the removal of *lon* reduces the virulence of *S*. Enteritidis even for highly sensitive Balb/C mice [17] and the production of mucoid colonies due to the overproduction of capsular polysaccharides may enable simple differentiation of the vaccine strain from those circulating in the environment. Finally, there are reports on the attenuation of *lon* mutants for chickens, originally for *S*. Gallinarum and recently also for *S*. Enteritidis [26]–[29]. In this study, we have therefore constructed a triple SPI1-*lon*-*fliC* mutant of *S*. Enteritidis and tested its efficacy as a live attenuated marker vaccine for the oral vaccination of poultry.

## Results

### Vaccine Strain Characterisation

**Figure 1:**
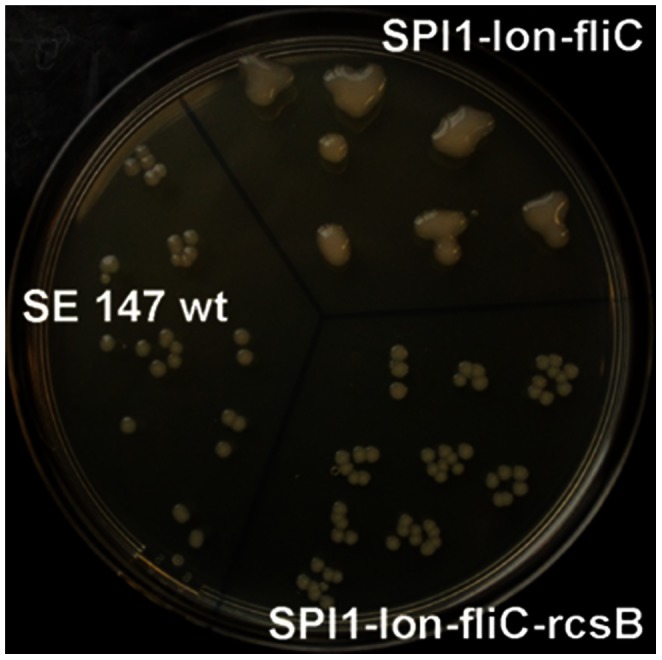
Colony morphology of the wild-type *S*. Enteritidis, SPI1- *lon*::Cm-*fliC* mutant and SPI1-*lon*::Cm-*fliC*-*rcsB*::Kan mutant. Inactivation of *lon* resulted in a mucoid colony phenotype which was observed in all the mutants with the *lon* mutation except for the mutant in which the *rcsB* mutation has been introduced. The overproduction of capsular polysaccharides in the vaccine strain enables simple differentiation of the vaccine strain from those circulating in the environment.

**Figure 2:**
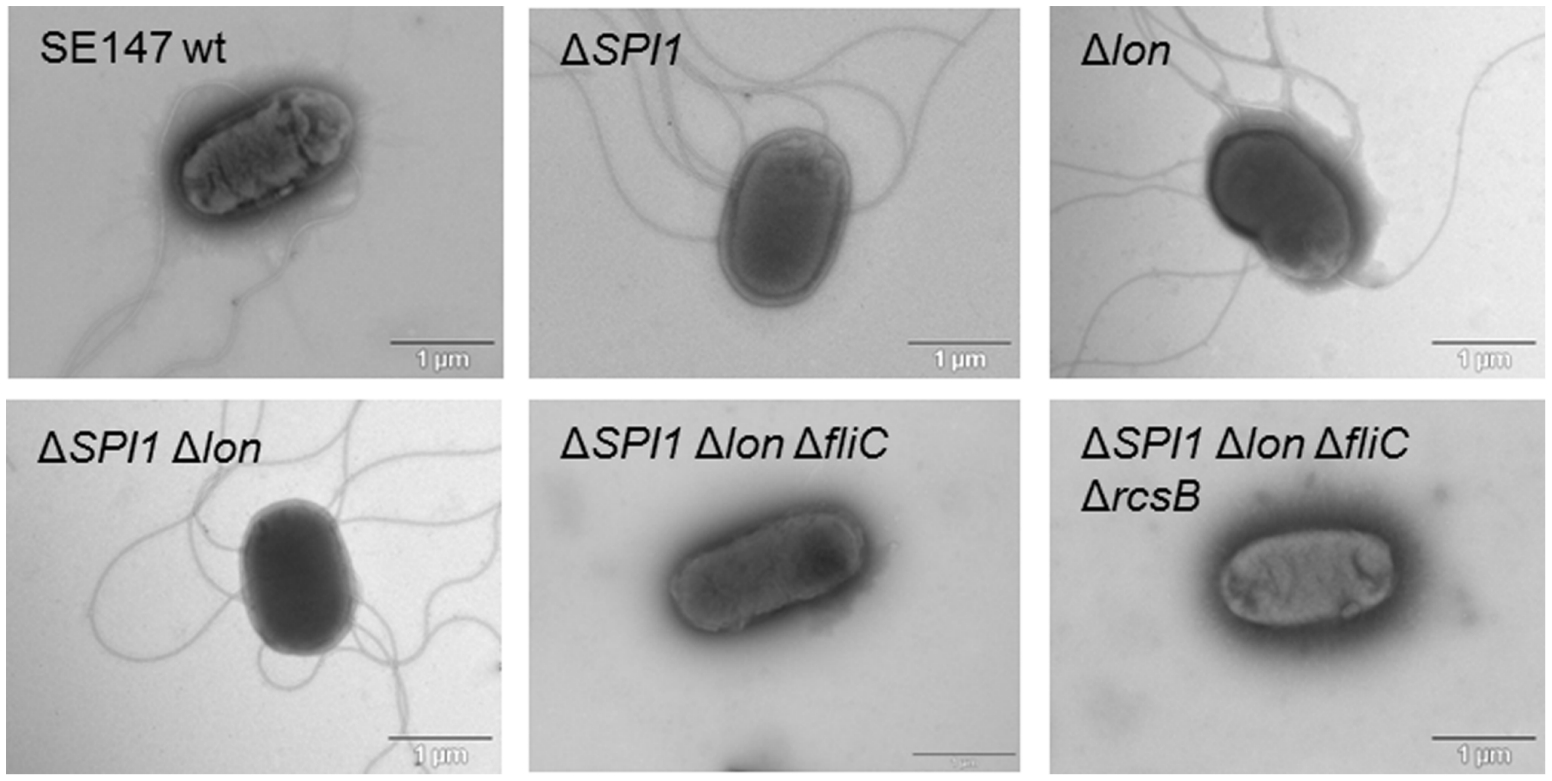
Electron microscopy of flagella in *S*. Enteritidis. Flagella could be visualised in all the strains and mutants with intact *fliC* after negative staining with ammonium molybdate.

Inactivation of *lon* resulted in a mucoid colony phenotype which was observed in all the *lon* mutants except for the SPI1-*lon*::Cm-*fliC*-*rcsB*::Kan mutant (Fig. 1). All the mutants harboring the *fliC* mutation were free of flagella on their surface (Fig. 2) and non-motile when inoculated in semisolid 0.3% agar (not shown).

### Experiment 1, Vaccination with SPI1 and *lon* Single Mutants

The protective capacity of the SPI1 and *lon* mutants for chickens was tested in the first vaccination trial. Three weeks after the first vaccination on the day of hatching, the SPI1 mutant efficiently colonized both the liver and caecum. After revaccination and prior to challenge on day 42 of life, the birds vaccinated with the SPI1 mutant were free of the vaccine strain in the liver but half of the birds remained positive in the spleen and 1 out of 6 tested chickens was positive in the caecum. The *lon* mutant was isolated from the vaccinated chickens with a lower frequency than the SPI1 mutant at day 42 although this difference did not reach statistical significance (Table 1). Four days post challenge, the SPI1 and *lon* mutant vaccinated chickens were protected against colonization of the liver and spleen but not the caecum. Fourteen days post infection, a positive effect of vaccination was observed also in the caecum as significantly less chickens tested positive when compared with the non-vaccinated controls (Table 1).

**Table 1:** Persistence, attenuation and protective capacity of the SPI1 and *lon* mutants for chickens.

	day 21&			day 42			day 46			day 56		
vaccination	liver	spleen	caecum	liver	spleen	caecum	liver	spleen	caecum	liver	spleen	caecum
ΔSPI1	5/6#	n.d.∧	6/6	0/6	3/6	1/6	2/6	1/6*	5/6	0/6	0/6*	1/6*
Δ*lon*	1/6	n.d.	0/6	0/6	1/6	0/6	1/6*	0/6*	6/6	0/6	0/6*	2/6
non vaccinated	n.d.	n.d.	n.d.	n.d.	n.d.	n.d.	5/6	6/6	6/6	2/6	6/6	6/6

& data for days 21 and 42 of life indicate persistence of the vaccine strains, data for days 46 and 56 of life indicate colonization by the challenge wild-type *S*. Enteritidis.

# number of positive chickens**/**number of tested.

∧ n.d., not determined due to the small size of some of the spleens of 21-day-old chickens.

* significantly different from non-vaccinated controls by χ^2^ test at P<0.05.

### Experiment 2, Oral Vaccination with the SPI1-*lon*, SPI1-*lon-fliC* and SPI1-*lon-fliC-rcsB* Mutants

Although removal of SPI1 results in attenuation of *S*. Enteritidis for chickens [5], we hypothesized that the removal of *fliC* may increase its virulence [12]. That is why we combined both attenuating mutations, i.e. SPI1 and *lon*. However, as the *lon* mutants overproduce capsular polysaccharides, we suppressed the overproduction of a capsule by the introduction of the *rcsB* mutation into SPI1-*lon*-*fliC* mutant. All the constructed vaccine strains were then tested as attenuated vaccines.

At 4 DPI, chickens vaccinated with the SPI1-*lon*-*fliC* vaccine were protected against oral challenge with wild-type *S*. Enteritidis as only one chicken tested positive in the liver and none of the challenged chickens tested positive in the spleen or caecum. Vaccination with the remaining two mutants, i.e. the SPI1-*lon* mutant and the quadruple SPI1-*lon*-*fliC*-*rcsB* mutant did not prevent early caecum, liver and spleen colonization in the challenged chickens at 4 DPI (Table 2).

**Table 2:** Protective capacity of the SPI1-*lon*, SPI1-*lon*-*fliC* and SPI1-*lon*-*fliC*-*rcsA* mutants after oral-oral vaccination and oral or intravenous challenge in chickens.

	day 42 of life Challenge	4 DPI			14 DPI		
Vaccination		Liver	spleen	caecum	liver	spleen	caecum
SPI1-*lon*::Cm	oral	1/6#	2/6	5/6&	1/6	0/6	3/6
SPI1-*lon*::Cm -*fliC*		1/6	0/6	0/6	0/6	1/6	2/6
SPI1-*lon*::Cm-*fliC*-*rcsB*::Kan		4/6	3/6	6/6&	0/6	0/6	3/6
non-vaccinated		2/6	2/6	6/6&	4/6	4/6	6/6
SPI1-*lon*::Cm	intravenous	3.94±0.47*	5.20±0.24*	6/6	3/6	2.40±1.41*	2/6
SPI1-*lon*::Cm -*fliC*		2.92±1.37*	4.89±0.72*	6/6	5/6	3.29±1.07	2/6
SPI1-*lon*::Cm-*fliC*-*rcsB*::Kan		3.97±0.20*	5.34±0.25*	6/6	6/6	3.58±0.31*	4/6
non-vaccinated		4.71±0.39	6.68±0.42	6/6	5/5	4.16±0.38	5/5

# number of positive chickens**/**number of tested.

* t-test different from the non-vaccinated control chickens at P<0.05.

& χ^2^ test different from the chickens vaccinated with the SPI1-*lon*-*fliC* mutant in caecum at 4 DPI (P<0.05).

Fourteen days post infection, chickens vaccinated with any one of the vaccine strains exhibited protection as lower numbers of positive chickens were observed when compared with the non-vaccinated controls. The protective effect was observed mainly in the liver and spleen and, to a lesser extent, also in the caecum (Table 2).

Intravenous challenge resulted in extensive tissue colonization. At 4 DPI, all three tested vaccines significantly reduced the bacterial load in the liver and spleen but not in the caecum. Between 4 and 14 DPI, one chicken in the non-vaccinated group died. Besides this, an approx. 2 log decrease in counts of challenge *S*. Enteritidis was observed in all groups (Table 2). In comparison with the non-vaccinated chickens, significantly lower *S*. Enteritidis counts were observed in the spleens of chickens vaccinated with the SPI1-*lon* and SPI1-*lon*-*fliC*-*rcsB* mutants at 14 DPI.

### Experiment 3, Intravenous Vaccination with the SPI1-*lon* and SPI1-*lon-fliC* Mutants

In the last experiment we were interested whether we could further increase chicken immunity by an intravenous application of the vaccine strain after two oral vaccination doses. In addition, this experiment allowed us to demonstrate the absence of anti-flagellin antibodies in the chickens vaccinated with the mutants harboring the *fliC* mutation. To reduce the number of treated animals, this experiment was performed with only the SPI1-*lon* and SPI1-*lon*-*fliC* mutants as the quadruple SPI1-*lon*-*fliC*-*rcsB* mutant appeared as the least immunogenic in the previous experiment (Table 2).

Although the i.v. vaccinated chickens were protected against challenge both at 4 and 14 DPI when compared with the non-vaccinated chickens, no additional protection after intravenous re-vaccination followed by oral challenge was observed when compared with the chickens vaccinated only orally in experiment 2 (compare tabs. 2 and 3). However, when the intravenously re-vaccinated chickens were challenged via the i.v. route, approx. 10 times better protection was achieved when compared with the chickens vaccinated only orally, though such comparison must be considered with a certain care since the challenged chickens were not of the same age. The increase in protective capacity after i.v. vaccination was significant when SPI1-*lon* mutant was used for the vaccination but did not reach statistical significance when the SPI1-*lon*-*fliC* mutant was used for the vaccination. Similar to the oral vaccination only, an efficient protection from caecum colonization by the challenge strain was achieved after the oral/oral/i.v. mode of vaccination with the SPI1-*lon*-*fliC* mutant as early as 4 DPI (Table 3).

**Table 3:** Protective capacity of the SPI1-*lon* and SPI1-*lon*-*fliC* mutants after oral-oral-intravenous vaccination, followed by oral or intravenous challenge in chickens.

	4 DPV$			14 DPV			day 62of lifechallen.	4 DPI			14 DPI		
vaccination	liver	spleen	caecum	Liver	spleen	caecum		liver	spleen	caecum	liver	spleen	caecum
SPI1-*lon*	4/6#	4.42±0.36	0/6	2/6	5/6	0/6	Oral	2/6	5/6	1/6*	0/6	2/6	0/6
SPI1-*lon*-*fliC*	4/6	4.21±0.48	0/6	1/6	6/6	0/6		0/6*	3/6	2/6	0/5	1/5	0/5
non-vaccinated	n.d.	n.d.	n.d.	n.d.	n.d.	n.d.		6/6	6/6	6/6	2/6	3/6	0/6
SPI1-*lon*							i.v	1.94±0.94&	4.27±0.38&	4/6	4/6	2.25±1.26&	0/6
SPI1-*lon*-*fliC*								2.26±0.89&	4.49±0.41&	0/6*	1/6*	2.17±1.19&	0/6
non-vaccinated								4.73±0.70	6.50±0.56	5/6	6/6	4.06±0.67	1/6

$ DPV, days post intravenous vaccination.

# number of positive chickens**/**number of tested.

* χ^2^ test different from the non-vaccinated control chickens at P<0.05.

& t-test different from the non-vaccinated control chickens at P<0.05.

### Antibody Production after Infection in Experiment 2 and 3

**Figure 3:**
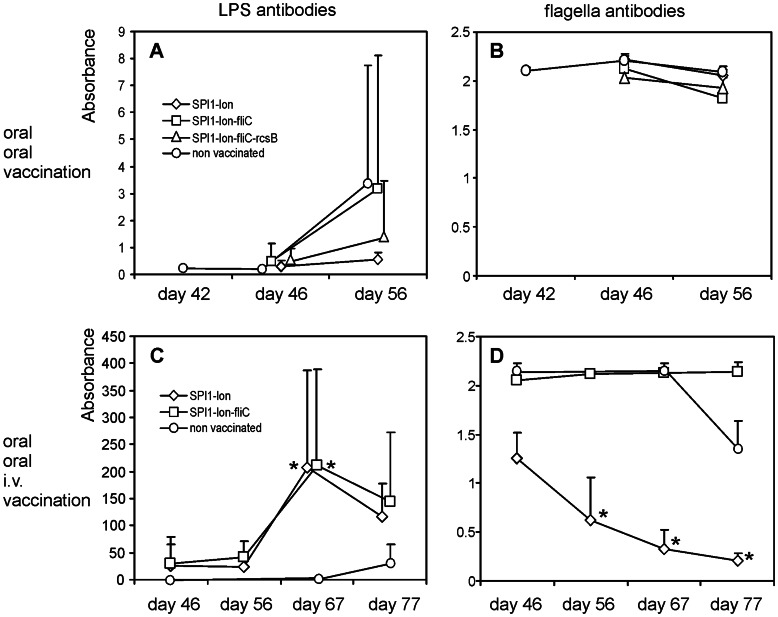
Antibody production in immunized and challenged chickens. Panel A, anti-LPS antibodies after oral vaccination and oral challenge on day 42 of the chicken’s life. Panel B, the same as in panel A except for the data shown for anti-flagellin antibodies. Panel C, anti LPS antibodies after oral vaccination and i.v. revaccination followed by oral challenge on day 63 of the chicken’s life. Panel D, the same as in panel C except for the data shown for anti-flagellin antibodies. As competitive ELISA was used, the increase in anti-flagellin antibody is characterized by a decrease in absorbance. Diamonds, SPI1-*lon*::Cm vaccinated chickens; squares, SPI1- *lon*::Cm-*fliC* vaccinated chickens; triangles, SPI1- *lon*::Cm-*fliC*-*rcsB*::Kan vaccinated chickens; circles, non-vaccinated chickens. * - significantly different from the non-infected controls sacrificed on day 42 by Kruskal-Wallis and post hoc Dunn’s test at P<0.05 (panels A and B) or day 46 (panels C and D).

Oral challenge in orally vaccinated chickens resulted in only a moderate antibody production. Anti-LPS antibodies increased weakly at 4 DPI in all groups of vaccinated chickens and the increase in antibody production continued up to 14 DPI. However, this increase was caused by two or three highly responding chickens what resulted in high within-group variation and insignificance statistical insignificance (Fig. 3A). Anti-flagellin antibodies were not produced by any of the orally vaccinated and orally challenged chickens, perhaps due to too short duration of the experiment (Fig. 3B).

Chickens vaccinated twice orally and revaccinated intravenously produced high levels of anti-LPS antibodies, independent of the vaccine strain used. The antibodies appeared as early as 4 days after the i.v. revaccination and reached statistical significance at 4 DPI when compared with the non-infected controls sacrificed on day 46 (Fig. 3). Chickens vaccinated with the SPI1-*lon*-*fliC* did not produce anti-flagellin antibodies at all, even after oral challenge with the wild type *S*. Enteritidis. On the other hand, anti-flagellin antibodies appeared in the group of chickens vaccinated with the SPI1-*lon* mutant 4 days after the i.v. revaccination and gradually increased as the experiment continued. Additionally, in this experiment we recorded the production of anti-flagellin antibodies also in the control group of non-vaccinated and orally challenged chickens at 14 DPI (Fig. 3).

## Discussion

The key characteristics for a new generation of live, attenuated *Salmonella* vaccine, besides the attenuation and immunogenicity, include an absence of any antibiotic resistance markers in the vaccine strain, the possibility of a simple vaccine strain differentiation and the possibility to differentiate vaccinated from naturally infected flocks [7], [9]. Except for the presence antibiotic resistance (chloramphenicol or kanamycine), vaccine strains described in this study provided all the remaining characteristics – and even the antibiotic resistance could be easily removed prior its commercial and widespread use.

Although we did not sacrifice orally vaccinated chickens on day 42 in experiments 2 and 3, and we therefore do not have data on antibody levels in these chickens, it is likely that these were very low because even 4 days after oral challenge with the wild type *S*. Enteritidis there were very low levels of anti-LPS or anti-flagellin antibodies. However, using intravenous vaccination we proved that the SPI1-*lon*-*fliC* mutant never induced production of anti-flagellin antibodies whilst these could be easily detected after intravenous vaccination with the SPI1-*lon* mutant. The use of the SPI1-*lon*-*fliC* mutant will therefore not result in anti-flagellin antibodies, which will enable the differentiation of vaccinated flocks from those naturally infected.

In the first experiment, we confirmed the protective capacity of the SPI1 and *lon* mutants of *S*. Enteritidis, as described previously for *S*. Gallinarum [27]. Based on these results we constructed 3 additional mutants. SPI1-*lon* and SPI1-*lon*-*fliC* mutants were designed to be of a similar attenuation differing only in their ability to stimulate the production of anti-flagellin antibodies. The third mutant SPI1-*lon*-*fliC*-*rcsB* was constructed to suppress the mucoid phenotype of the *lon* mutation. However, the SPI1-*lon*-*fliC*-*rcsB* mutant was the least protective, either due to an additional attenuation caused by the *rcsB* mutation [30], [31] or due to the suppression of the mucoid phenotype by capsule overproduction, which may increase the immunogenicity of the *lon* mutants. Indeed, the *lon* mutants, though attenuated, exhibit a prolonged persistence in mice [17].

When the immunogenicity SPI1-*lon* mutants, with or without intact *fliC* was compared, vaccination with the SPI1-*lon*-*fliC* mutant resulted in slightly more efficient protection of chickens than the vaccination with the SPI1-*lon* mutant. This might be related to the fact that flagellin is a ligand for TLR5. The vaccination with the flagellin-positive SPI1-*lon* mutant led to the production of anti-flagellin antibodies which may bind to flagellin of the challenge strain and prevent its recognition by TLR5 [14]. Chickens vaccinated by the SPI1-*lon* mutant therefore responded to *S*. Enteritidis challenge by a well-developed specific immune response, but unlike the SPI1-*lon*-*fliC* vaccinated chickens, perhaps without the activation of the TLR5-dependent innate immune response. A similar negative effect of anti-flagella antibodies to challenge has been reported in mice infected *S*. *enterica* or *Pseudomonas aeruginosa* [7], [15], [32].

## Materials and Methods

### Ethics Statement

The handling of animals in the study was performed in accordance with current Czech legislation (Animal protection and welfare Act No. 246/1992 Coll. of the Government of the Czech Republic). The specific experiments were approved by the Ethics Committee of the Veterinary Research Institute (permit number 48/2010) followed by the Committee for Animal Welfare of the Ministry of Agriculture of the Czech Republic (permit number MZe 1226).

### Bacterial Strains

*S*. Enteritidis 147 with proven virulence and ability to colonize the chicken gut was used [20]. The construction of the SPI1 mutant with the whole pathogenicity island SPI1 removed from the chromosome has been described earlier [20], [33]. *lon*::Cm, *fliC*::Cm and *rcsB*::Kan mutations were constructed by λ red recombination [34] and transferred to final recipients by P22-mediated transduction [20]. Each of the mutation was verified by PCR and primer pairs used for the construction of *lon*::Cm, *fliC*::Cm and *rcsB*::Kan mutations and PCR verifications are listed in Table 4. After each transduction, the resulting transductant was checked for sensitivity to P22 phage and, if necessary, the chloramphenicol gene cassette was excised from the chromosome by transient transformation with plasmid pCP20 [34]. Genotypes of the resulting mutants therefore were ΔSPI1, Δ*lon*, ΔSPI1 *lon*::Cm, ΔSPI1 *lon*::Cm Δ*fliC* and ΔSPI1 *lon*::Cm Δ*fliC rcsB*::Kan. To simplify enumeration, the wild-type *S*. Enteritidis and all mutants were spontaneously resistant to nalidixic acid which, to our best knowledge, does not affect this strain virulence.

**Table 4:** List of primers used in this study for the construction of *fliC*, *rcsB* and *lon* mutants.

Name*	Primer 5′-3′
fliC_44F	GTCGGTGAATCAATCGCCGGATTAACGCAGTAAAGAGAGGACGT
fliC_44R	AGTCATTAATACAAACAGCCTGTCGCTGTTGACCCAGAATAACC
rcsB_44F	ATGAACAATATGAACGTAATTATTGCCGATGACCACCCGATTGT
rcsB_44R	TTATTCTTTGTCTGTCGGACTCAGGGTGACAGAAGAGAGATAGT
lon_51F	CAGCTATACTATCTGATTACCTGGCGGACACTAAACTAAGAGAGAGCTCTT
lon_50R	CGAAATAGCCTGCCAGCCCTGTTTTTATTAGCGCTATTTGCGCGAGGTCA
fliC_FCTR	TGGCGAGATATTTTTTAACC
fliC_RCTR	AGTAGTTAAGCGCGTTATCG
rcsB_FCTR	GGCTATTATGCGCTATTTGT
rcsB_RCTR	ATATTGTTCTGAGCGATGTG
lon_FCTR	GCAGGCTTCTGGCGAATAAT
lon_RCTR	CGACCGCGCAGCAGTTATAT

* For primers used for the amplification of pKD3 or pKD4, only the gene specific overhangs are shown. „CTR“ primers, either Forward (F) or Reverse (R) were used for the verification of the final contructs.

### Experimental Animals

Male, newly-hatched ISA Brown Chickens (Hendrix Genetics, Boxmeer, The Netherlands) were used in this study. The chickens were reared in perforated plastic boxes with free access to water and feed. Each of the experimental or control groups was kept in a separate room.

### Experimental Design

In the first vaccination trial (Experiment 1), 60 chickens were divided into 2 experimental groups of 24 chickens each (group 1 and 2), and a control group of 12 non-vaccinated chickens (group 3). Group 1 was orally vaccinated with the SPI1 mutant and group 2 with the *lon* mutant. The chickens were vaccinated orally on day 1 of life and revaccinated on day 21 with 10^7^ CFU of appropriate vaccine strain per chicken. On days 21 and 42, 6 vaccinated chickens from each group were sacrificed and the remaining chickens were orally challenged with 3×10^7^ CFU of the wild type *S*. Enteritidis in LB broth. Six birds from each group were euthanized 4 and 14 days post infection (DPI), respectively.

In the second vaccination trial (Experiment 2), 102 chickens were divided into 3 experimental groups of 24 birds each (group 1, 2 and 3), and a control group of 30 non-vaccinated chickens (group 4). Group 1 was orally vaccinated with the SPI1-*lon* mutant, group 2 with the SPI1-*lon*-*fliC* mutant and group 3 with SPI1-*lon*-*fliC*-*rcsB* mutant. The chickens were vaccinated on day 1 of life and revaccinated on day 21 with 10^7^ CFU of appropriate vaccine strain per chicken in LB broth. On day 42, 6 non-vaccinated chickens were sacrificed and the remaining chickens in each group were challenged with wild type *S*. Enteritidis. Half of the chickens were challenged orally with 3×10^7^ CFU of *S*. Enteritidis in LB broth and the remaining half were intravenously challenged with 10^7^ CFU of *S*. Enteritidis in 0.1 ml of PBS.

Six birds from each group were euthanized 4 and 14 DPI, respectively. The intravenous challenge in experiment 2 and experiment 3 (see below) was performed to assess the resistance of the vaccinated birds to an extreme level of systemic infection and to get strong serological response to LPS and flagella.

In the last vaccination trial (Experiment 3), 102 chickens were divided into 2 experimental groups of 36 birds each (group 1 and 2), and a control group of 30 non-vaccinated chickens (group 3). Group 1 was vaccinated with the SPI1-*lon* mutant and group 2 with the SPI1-*lon-fliC* mutant. The chickens in group 1 and 2 were orally vaccinated on day 1 of life, orally revaccinated on day 21 and intravenously revaccinated on day 42 with 10^7^ CFU of appropriate vaccine strain per chicken. The chickens in group 3 served as non-vaccinated controls. On day 63, the chickens were either orally or intravenously challenged as described above and 6 birds from each group were euthanized 4 and 14 DPI, respectively.

### Sample Collection and Processing

At the end of each experiment, blood from each bird was collected for serological tests and samples of the liver, spleen and cecal content were processed for enumeration of *S*. Enteritidis. These samples were homogenized in peptone water, tenfold serially diluted and plated on XLD agar plates (HiMedia) supplemented with 20 µg/ml nalidixic acid. Detection limit of direct plating was 500 CFU/g of sample. Samples negative after direct plating were subjected to enrichment in modified semi-solid Rappaport-Vassiliadis medium (Oxoid) for qualitative *S*. Enteritidis determination. Counts of *S*. Enteritidis positive after direct plating were logarithmically transformed. Samples positive only after enrichment were assigned a value of 1 and negative samples were assigned a value of 0.

### ELISA Detection of Anti-LPS and Flagella Antibodies

A commercial FLOCKSCREEN™ *Salmonella* Enteritidis Antibody ELISA kit (x-OvO Limited) was used for the detection of anti-LPS serum antibodies. For anti-flagella antibodies, a FlockCheck kit was used as recommended by the manufacturer (IDEXX Laboratories, USA). Both ELISA tests were performed as recommended by the manufacturers except that the sera were diluted from 1∶10 up to 1∶8000 using sample dilution buffer to reach the absorbance which could be measured by the spectrophotometer, i.e. ranging from 0.2 to 1.8. The real absorbances were then calculated knowing the read absorbance and particular dilution, and such data are used throughout this study.

### Transmission Electron Microscopy

A formvar-coated copper grid was placed on a single drop of overnight culture for 5 min. The grid was washed twice in a drop of water, stained with 1% ammonium molybdate and observed with a Philips EM 208 transmission electron microscope under an acceleration of 80 kV.

### Statistical Analysis

The χ^2^ square test and Student’s t-test were used for bacteria counts analysis as indicated in the text. Antibody response was analysed by Kruskal-Wallis test followed by post hoc Dunn’s test. SPSS v.14 software was used for statistical calculations.
